# Amnion-derived Cellular Cytokine Solution: A Physiological Combination of Cytokines for Wound Healing

**Published:** 2008-04-07

**Authors:** David L. Steed, Cathy Trumpower, Danelle Duffy, Charlotte Smith, Vivienne Marshall, Randall Rupp, Martin Robson

**Affiliations:** University of Pittsburgh/UPMC, Pittsburgh, PA;; Stemnion, Inc, Pittsburgh, PA; University of South Florida, Tampa, FL; and; Institute for Tissue Regeneration, Repair, Rehabilitation, Bay Pines VA Medical Center, Bay Pines, FL

## Abstract

**Objective:** Wound repair is a complex integration of dynamic processes mediated by humeral messages controlling the levels of cytokines, growth factors, and matrix metalloproteinases in the wound space. Isolated growth factors and growth factor combinations have been used to accelerate wound healing with limited success. A cellular cytokine solution can be collected by harvesting the proteins released from amnion-derived multipotent progenitor cells. The purpose of this study was to compare levels of cytokines/growth factors in amnion-derived cellular cytokine solution with physiological levels reported in the medical literature. **Methods:** Amnion-derived multipotent progenitor cells were grown to confluency, and the proteins secreted were characterized by qualitative and quantitative analysis. These results were compared with physiologic levels reported in the medical literature. **Results:** The results demonstrated that amnion-derived cellular cytokine solution contained physiologic levels of cytokines relevant to wound healing, including platelet-derived growth factor, vascular endothelial growth factor, angiogenin, transforming growth factor *β* 2, tissue inhibitor of metalloproteinase-1, and tissue inhibitor of metalloproteinase-2. The ranges (mean ± standard deviation) were as follows: platelet-derived growth factor, 86 ± 33 pg/mL; vascular endothelial growth factor, 5.7 ± 1.5 ng/mL; angiogenin, 1.0 ± 0.33 ng/mL; transforming growth factor *β* 2, 500 ± 330pg/mL; tissue inhibitor of metalloproteinase-1, 530 ± 140 ng/mL; and tissue inhibitor of metalloproteinase-2230 ± 40 ng/mL. These levels are comparable with the physiologic levels reported in the literature. **Conclusions:** The physiologic levels of cytokines important to healing found in amnion-derived cellular cytokine solution suggest that amnion-derived cellular cytokine solution may be of benefit in healing certain acute and chronic wounds.

Wound repair is a complex integration of dynamic interactive processes involving cell-cell and cell-matrix interactions mediated by humoral messengers.^1^ These messengers that control the various cellular processes include cytokines, growth factors, and matrix metalloproteinases (MMPs).^2^ They regulate many of the processes that are crucial for wound healing, including chemotactic migration of inflammatory cells; mitosis of fibroblasts, keratinocytes, and vascular endothelial cells; neovascularization; and synthesis and degradation of extracellular components.^3^

The literature is replete with examples of effects of exogenous application of cytokines in animal models of both acute and chronic wounds.^4^ In all of these animal models, it has been suggested that wound healing would be enhanced by topical application of cytokines. Clinical trials have been performed for many of the cytokines and/or growth factors in both acute and chronic wounds.^4^ Unfortunately, the results of these trials have been largely disappointing, with only a single growth factor, platelet-derived growth factor (PDGF)-BB, being approved by the US Food and Drug Administration, and that was for a single indication, diabetic foot ulcers.

There are several reasons why topical cytokine/growth factor therapy has failed.^5^ These include application of a single growth factor, application in large pharmaceutical doses, degradation of the peptide by proteases, and improper preparation of the wound. The application of a single growth factor may seem ill conceived. Normal wound healing is accomplished by a combination of cytokines, and they occur in a “natural“ cascade.^6^ Only a few attempts of combination or sequential cytokine therapy have been reported. Knighton et al^7^ used an autologous platelet releasate labeled as platelet-derived wound healing factors (PDWHF). Steed et al^8^ reported similar success with PDWHF. An extract of milk that contained insulin-like growth factor-1, PDGF, basic fibroblast growth factor, and transforming growth factor *β* (TGF-*β*) has been reported to be effective in accelerating healing inanimal models.^9^ The first wound healing clinical trial using sequential topically applied cytokines to accelerate healing was reported in 2000.^10^

The possible combinations or sequences for cytokines are endless. Moreover, attempting to determine levels of the various factors to include is formidable. The concept behind PDWHF was that the platelet releasate contained its cytokines in physiologic levels. Unfortunately, these levels were never critically examined, nor determined to be physiologic. Stem cells and stem cell–like multipotent cells are known to produce cytokine growth factors that serve as mediators to the cellular processes of the wound healing scheme.^11^ In a recent article, Xing et al^12^ demonstrated that amnion-derived multipotent progenitor cells increased gain of incisional breaking strength and decreased the incidence and severity of acute wound failure.^12^ They postulated that 1 possible mechanism for their results could be the effect of the cocktail of secreted cytokines from the cells.

Cells in culture secrete factors, which may provide support to or affect the growth, differentiation, and protein production of other cells. These factors include cytokines, growth factors, chemokines, hormones, proteins, extracellular matrix, vesicles, receptors, antibodies, inhibitors, and granules. The purpose of this study was to determine the presence and levels of cytokines/growth factors in amnion-derived cellular cytokine solution (ACCS) and review the literature to determine the natural or physiological levels of these peptides in normally healing wounds.

## MATERIALS AND METHODS

Full-term placentas donated for research using an “honest broker” system to maintain anonymity were obtained from scheduled caesarian section deliveries with hospital approval. All placentas were devoid of identifiers when received. Amnion-derived multipotent progenitor cells were grown to confluency, and using proprietary techniques, the supernatant was harvested. This secreted product was labeled ACCS. The proteins in the ACCS were characterized by both qualitative and quantitative analysis.

### Qualitative analysis

ACCS samples were shipped overnight on dry ice to RayBiotech (Norcross, Ga) for analysis in the Human Growth Factor Antibody Array 1, Human Matrix Metalloproteinase Antibody Array 1, and Human Cytokine Antibody Arrays 6, 7, and 8.

### Quantitative analysis

ACCS samples were shipped overnight on dry ice to Pierce Biotechnology (Woburn, Mass) for analysis in the Multiplex Custom Searchlight Human Protein Antibody Array.

Samples were also sent to RayBiotech (Norcross, Ga) for analysis in the Custom Human Cytokine Antibody Array. In-house quantitative measurements were conducted using RayBiotech's RayBio Human ELISA kits for the cytokines VEGF, PDGF-BB, angiogenin, TGF-*β* 2, TIMP-1, and TIMP-2. Detectable limits of the assays were the following: VEGF, 20 pg/mL; PDGF-BB, 2 pg/mL; angiogenin, 1.5 pg/mL; TGF-*β* 2, 15 pg/mL; TIMP-1, 40pg/mL; and TIMP-2, 10 pg/mL. ACCS samples were assayed in duplicate. Results were in the linear portion of the standard curve. Other cytokines and growth factors were detected but not quantified.

To determine the physiologic levels of cytokines and growth factors in humans, the literature was reviewed by searching PubMed, MEDLINE, and the Cochrane Database of Systematic Reviews.

## RESULTS

ACCS was characterized by assaying for physiologically relevant cytokines, including PDGF, VEGF, angiogenin, TGF-*β* 2, TIMP-1, and TIMP-2. Cytokines are secreted from these cells in the physiologic range reported in the literature.

Results are as follows (see Fig 2):

**Figure:**
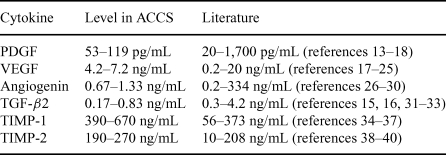

## LITERATURE REVIEW

PDGF levels have been measured and reported in many different fluids in both normal and diseased tissues. Some reports measure total PDGF, whereas others measure the PDGF-BB homodimer or the PDGF-AB heterodimer. In most studies, PDGF was present in picograms per milliliter, although some studies found amounts in nanogram. The physiologic range chosen here was from studies sampling serum in healthy patients (1700 pg/mL),^13^ normal sinus fluid (311 pg/mL),^14^ healing and nonhealing ankle ulcers (133 pg/mL and 97 pg/mL),^15^ burn blister fluid (154 pg/mL),^16^ and surgical drains (20–180 pg/mL)^17^ and wound drainage following colorectal procedures (817 pg/mL, 74–1015 pg/mL).^18^ PDGF-BB level in ACCS is within the normal physiologic range reported in the literature.

VEGF levels were also measured in many different tissues, both healthy and diseased, at many different time points. In most cases, the levels were in amounts of nanograms per milliliter. VEGF level was low in serum (0.2 ng/mL).^19^ This would be anticipated because there is little reason physiologically why an angiogenesis factor should circulate through the blood at an elevated level under normal circumstances. VEGF levels were the same in drainage from breast wounds, whether benign or malignant disease (1–3.1 ng/mL).^18^,^20^ Similar levels of VEGF were found in fluid from hernia repairs, surgical drains, and seromas (1.2–20 ng/mL),^17^,^21^ as well as in wounds following colorectal surgery (0.8–2.8 ng/mL)^22^ and thyroid cysts which spontaneously regressed (4.3 ng/mL).^23^ In most cases, VEGF levels were similar to ACCS. Very high levels of VEGF were found in wound fluid following hernia repair with mesh (160–330 ng/mL),^24^ but these levels were not considered physiologic and not included in the table because they may have been associated with the prosthetic material. VEGF level was also much higher in the granulation tissue of healing sacral decubitus ulcers, well above the physiologic range (1100 ng/mL)^25^.

Angiogenin levels were measured in a control population of several different studies. Levels of angiogenin in ACCS were at the lower end of the physiologic ranges reported (0.2–334 ng/mL).^26^–^29^ Angiogenin levels were measured in amniotic fluid and were greater than ACCS (22–32 ng/mL).^30^

TGF-*β* 2 plays a critical role in the healing process and scarring. TGF-*β* 2 was measured in split-thickness skin grafts (0.03 ng/mL)^31^ and in healing and nonhealing wounds (0.02–0.03 ng/mL).^15^ These levels were about the same in burn blisters (0.04 ng/mL)^16^ and breast cysts (0.1–0.3 ng/mL).^32^ In each of these cases, the levels of TGF-*β* 2 measured were in the range found in ACCS. The levels of TGF-*β* 2 were much high in the wound fluid of patients following reduction mammoplasty, an operation with much soft tissue dissection (16–38 ng/mL).^33^

TIMP-1 and TIMP-2 have been measured as controls in 2 studies (373 ng/mL and 106 ng/mL).^34^,^35^ TIMP-1 level has been shown to be higher in patients with pressure ulcers characterized as “good healers” (170 ng/mL)^36^ than “poor healers” (56 ng/mL).^36^ TIMP-1 level is much higher in wound drainage when negative pressure wound therapy is used (1493 ng/mL),^37^ perhaps related again to stimulation by foreign body. For this reason, this level was not considered to be physiologic. TIMP-1 levels in ACCS are at the upper limit of normal or somewhat above. TIMP-2 level was much higher in healthy controls (49 ng/mL and 136–208 ng/mL)^38^,^39^ than patients with diabetes (10 ng/mL).^40^ Thus, ACCS levels of both TIMP-1 and TIMP-2 are at the upper limit of the physiologic range and above.

## DISCUSSION

The purpose of this work was to determine the levels of certain cytokines or growth factors released into the supernatant of cultures of amnion-derived multipotent progenitor cells. These levels were then compared with physiologic levels measured in humans and reported in the literature. An extensive review of the literature yielded very few direct measurements of cytokines or growth factors in normal tissues.

The data that are available, in general, and are reported for individual cytokines or growth factors isolated from a single site. A physiologic growth factor solution containing a combination of growth factors has been reported as PDWHF. These growth factors have been harvested from platelets by centrifugation of platelets from whole blood, then releasing the contents of the *α*-granules of the platelet with thrombin. The “platelet releasate” was used clinically by diluting the growth factor preparation with buffered saline. The concentration of growth factors is not “physiologic” in contrast with the concentrations measured in ACCS. The concentration in PDWHF depends on the amount of buffered saline added to the releasate. There have been several reports of successful treatment of lower-extremity wounds with PDWHF, but the concentration of growth factors was much higher than the physiologic concentration measured in ACCS.^7^,^8^

One of the growth factors in ACCS, PDGF-BB, has been made through a recombinant DNA technique. It has been used in the healing of diabetic foot ulcers in randomized prospective blinded trials and found to be of benefit. It is marketed as becaplermin (Regranex). Packaged in a concentration of 0.1% or 0.1 mg/mL, PDGF is present in 1 million times the concentration found in ACCS. Of note, there have been no reported cases of toxicity from systemic absorption of becaplermin and no antibodies to becaplermin were found in patients exposed to this drug applied topically.

VEGF, angiogenin, and to some degree, PDGF, are angiogenesis factors. VEGF is a signaling protein involved in stimulating the growth of new blood vessels. In hypoxia, cells may produce hypoxia-inducible factor, which stimulates the release of VEGF, which binds to VEGF receptors, in turn leading to angiogenesis. Angiogenesis is essential to wound healing in every clinical setting. Angiogenin is also a polypeptide involved in angiogenesis. It triggers angiogenesis by inducing the proliferation of endothelial cells. PDGF regulates the growth and replication of cells and, as such, plays a pivotal role in wound healing. It stimulates angiogenesis but plays a role in many other functions including cell proliferation and cell migration. Angiogenesis factors may promote tumor growth; VEGF and angiogenin levels are low in ACCS.

TGF-*β* controls proliferation and differentiation of cells. Many cells make TGF-*β* and most have receptors for this protein. TGF-*β* plays a role in hormone secretion and wound healing.

TIMPs are natural inhibitors of MMPs, a group of peptidases, which degrade extracellular matrix. MMPs degrade growth factors; TIMPs counteract that process. TIMP also promotes cell proliferation and many prevent apoptosis. TIMP-1 binds to proMMP-9 and TIMP-2 binds to proMMP-2. MMPs have been shown to inhibit healing and degrade growth factors. Inhibition or inactivation of MMPs should facilitate healing.

The study of normal cytokine levels in animals is necessary if one is to determine the direction and magnitude of change of these proteins in pathological conditions. Tissues can be sampled and animals can be sacrificed to allow for these measurements. Invasive monitoring and sampling can be performed with little regard, to some degree, for sterility and the consequences to the laboratory animal. These issues become critical when attempting to measure the cytokine levels in humans. First, there is the issue of scientific merit. An investigator must convince himself or herself, the patient, and the institutional review board that the risk to the individual of measuring these levels is justified. The challenges are a bit different when one attempts to measure these levels in disease states. The levels can be determined by the study of tissue, blood, body fluids, tumors, cysts, wounds, seromas, and wound drainage. Cytokines can be measured in both the pathological tissue and the normal tissue adjacent to the abnormality. The issue in this circumstance is, of course, whether the neighboring tissue in a diseased patient is truly normal.

If one samples over time, cytokine levels change. The question then becomes what measurement is the physiological value versus a pathophysiological level.

All of the issues discussed here were encountered when attempting to determine the physiologic levels of cytokines to compare with the levels secreted by amnion-derived multipotent progenitor cells. The first issue is which of the many cytokines known to be present should be studied in depth. Some of the factors measured in ACCS are known to be important to wound healing. These include PDGF, VEGF, angiogenin, TGF-*β* 2, TIMP-1, and TIMP-2. The levels were measured by antibody array, enzyme-linked immunosorbent assay, and multiplex and mass spectroscopy.

Each of the techniques yielded a somewhat different result. Differences in culture techniques and conditions also lead to some differences in cytokine levels. The measurements were, however, within the same magnitude even when measured by different methods.

The greater challenge was to decide what constituted a “physiologic” level. Cytokines and growth factor levels were measured in normal tissues including blood. In some cases, the “normal” tissue levels were taken from patients with illnesses—were these truly normal? In other cases, the levels were taken from diseased tissues, but most were from normal physiologic states and processes including wounds, wound drainage, cysts, and fluid collections such as seromas. Moreover, the levels were measured at different stages of wound healing in patients with problems of different severity. Our approach was to decide the “normal or physiologic range” by looking for normal tissues or tissues closest to normal in patients with the least amount of problems.

There was reasonably good correlation between levels of cytokines measured in humans and the level of cytokines found to be secreted by the amnion-derived multipotent progenitor cells. In most cases, the levels measured in ACCS were near the lower end of the physiologic range, except for TIMP-1 and TIMP-2, which were at the upper limit. These physiologic levels of cytokines important in wound healing lead us to believe that an ACCS may be of benefit in healing acute and chronic wounds when used in a context of good clinical care.

## ACKNOWLEDGMENTS

This study was supported by Stemnion, Inc, Pittsburgh, Pennsylvania.

## Figures and Tables

**Figure 1:**
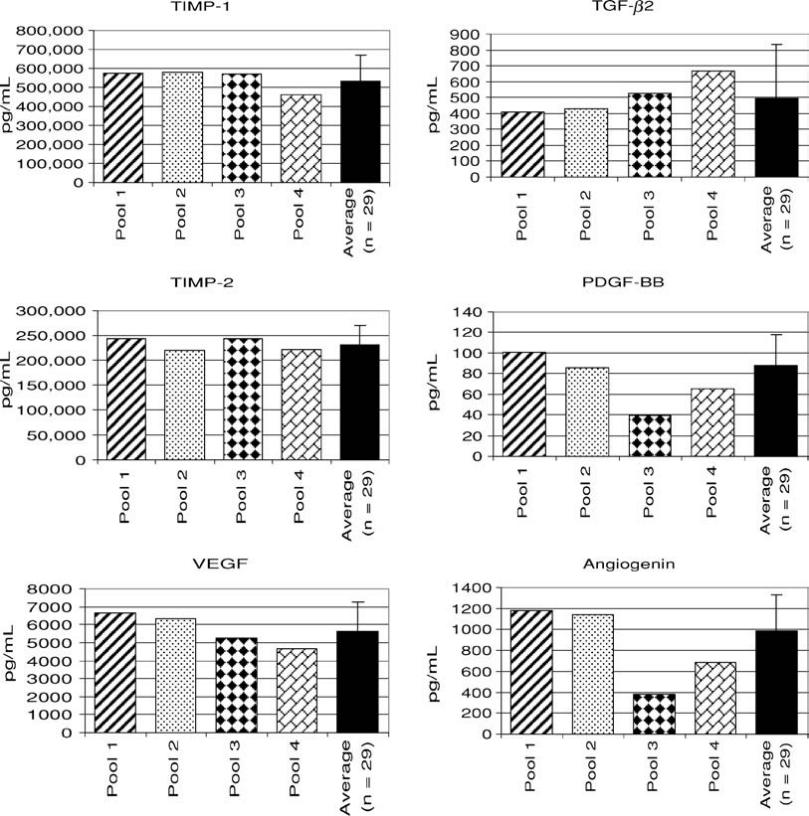
Cytokine, levels in amnion-derived cellular cytokine solution (ACCS) literature. Pools are made up of 9 (pool 1), 8 (pools 2 and 4), or 4 (pool 3) individual ACCS harvests. The solid bar shows the average and SD of the ACCS harvests for the 29 individual placentas contributing to the pools. TIMP-1 indicates tissue inhibitor of metalloproteinase-1; TGF-*β* 2, transforming growth factor *β* 2; TIMP-2, indicates tissue inhibitor of metalloproteinase-2; PDGF-BB, platelet-derived growth factor-BB; and VEGF, vascular endothelial growth factor.
